# Harnessing the Power of the Gut Microbiome: A Review of Supplementation Therapies for Metabolic Syndrome

**DOI:** 10.7759/cureus.69682

**Published:** 2024-09-18

**Authors:** Nikhil Reddy, Anjalee Chiwhane, Sourya Acharya, Sunil Kumar, Avinash Parepalli, Manikanta Nelakuditi

**Affiliations:** 1 Internal Medicine, Jawaharlal Nehru Medical College, Datta Meghe Institute of Higher Education & Research, Wardha, IND

**Keywords:** dysbiosis, gut microbiome, insulin resistance, metabolic syndrome, probiotics, supplementation therapies

## Abstract

Metabolic syndrome (MetS) is a complex condition characterized by abdominal obesity, insulin resistance, dyslipidemia, and hypertension, all of which increase the risk of cardiovascular disease and type 2 diabetes. The gut microbiome plays a significant role in metabolic health, influencing digestion, immune function, and energy metabolism. When the gut microbiota becomes imbalanced due to poor diet and antibiotic use, it can lead to systemic inflammation, insulin resistance, and abnormal lipid metabolism, which are central features of MetS. This review explores the connection between gut microbial imbalances and MetS, focusing on the impact of the gut microbiome on metabolic health. Supplementation therapies targeting the gut microbiome, such as probiotics, prebiotics, synbiotics, and postbiotics, are evaluated for their potential to improve metabolic parameters in MetS patients. These interventions hold promise for enhancing insulin sensitivity, reducing inflammation, and improving lipid profiles. However, further research is needed to optimize these approaches for managing MetS. Understanding how to leverage the gut microbiome could lead to innovative, non-invasive treatments for this growing global health concern.

## Introduction and background

Metabolic syndrome (MetS) is a cluster of interconnected metabolic disorders that significantly raise the risk of cardiovascular disease, type 2 diabetes, and other chronic health conditions [1]. The syndrome is diagnosed based on the presence of at least three key risk factors: abdominal obesity, hypertension, dyslipidemia, insulin resistance, and elevated fasting glucose levels. Together, these components increase the likelihood of atherosclerosis, systemic inflammation, and overall metabolic dysfunction [2]. Among these factors, central or abdominal obesity is particularly critical, often serving as the central driver of MetS due to its role in promoting insulin resistance and inflammation [3]. Globally, the prevalence of MetS has surged in recent decades, largely fueled by the growing rates of obesity and sedentary lifestyles. Currently, MetS affects around 25% of adults worldwide, with higher rates observed in developed countries where diets rich in processed foods and sugar are coupled with reduced physical activity [4]. The rising prevalence of MetS poses a significant public health challenge, as it accelerates the onset of chronic diseases, reduces life expectancy, and increases healthcare costs. The pathophysiology of MetS is complex, involving both genetic and environmental factors. Insulin resistance, a key feature of MetS, prevents cells from efficiently using glucose for energy, leading to elevated blood sugar levels and compensatory increases in insulin production [5]. Factors such as poor diet, physical inactivity, and exposure to environmental toxins also exacerbate the risk of developing MetS. While lifestyle modifications remain the cornerstone of treatment, recent research into the gut microbiome has revealed promising new strategies for managing MetS [6].

The human gut is home to trillions of microorganisms, collectively called gut microbiota. These microbes, which include bacteria, viruses, fungi, and archaea, play a vital role in maintaining overall health by regulating digestion, immune responses, and metabolism [7]. The composition and diversity of the gut microbiota vary widely between individuals and are influenced by genetics, diet, age, and antibiotic exposure. A healthy microbiome is typically diverse, with a balance of beneficial bacteria such as Bifidobacterium and Lactobacillus promoting optimal gut function [8]. One of the primary functions of the gut microbiota is to assist in the digestion of complex carbohydrates and fibers that are indigestible by human enzymes. These bacteria ferment these compounds into short-chain fatty acids (SCFAs) such as butyrate, acetate, and propionate, which serve as key energy sources for the host and possess anti-inflammatory properties [9]. Beyond digestion, the gut microbiota plays a crucial role in regulating the immune system, enhancing the gut barrier function, and preventing harmful pathogens from entering the bloodstream [10]. When the composition of the microbiota becomes imbalanced, a condition known as dysbiosis, it can lead to adverse health effects, including increased intestinal permeability, commonly referred to as "leaky gut." This allows endotoxins like lipopolysaccharides (LPS) to enter the bloodstream, triggering systemic inflammation and contributing to insulin resistance. Dysbiosis also affects fat storage, energy metabolism, and glucose regulation, all of which are key components in the development and progression of MetS [10].

As evidence linking the gut microbiota to metabolic health continues to grow, understanding how gut dysbiosis contributes to MetS has become a critical area of research. This review explores the relationship between the gut microbiome and MetS, highlighting the mechanisms through which microbial imbalances influence the metabolic disturbances associated with the syndrome. This review will assess the efficacy of current supplementation strategies aimed at modulating the gut microbiota, including probiotics, prebiotics, synbiotics, and postbiotics. It will examine their impact on key metabolic parameters such as insulin sensitivity, lipid profiles, and inflammation in individuals with MetS. By synthesizing the available literature, this review aims to provide insights into the therapeutic potential of gut microbiome-targeted interventions for managing MetS while also identifying areas for future research and clinical application.

## Review

Pathophysiology of metabolic syndrome and gut dysbiosis

**Figure 1:**
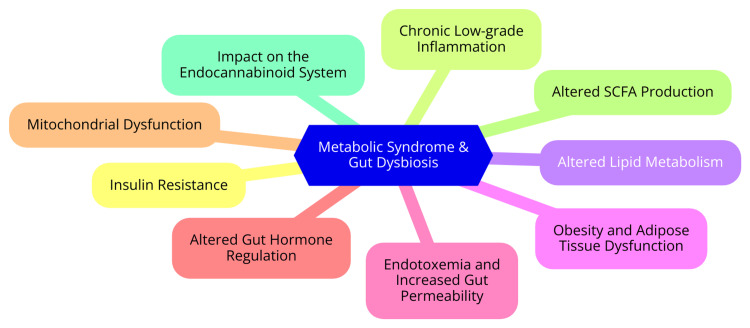
The pathophysiology of metabolic syndrome and gut dysbiosis. Image Credit: Dr. Nikhil Reddy.

The relationship between metabolic syndrome (MetS) and gut dysbiosis is an emerging field of research, highlighting the impact of gut microbiota alterations on obesity, insulin resistance, and inflammation [11]. Gut dysbiosis, defined as an imbalance in the gut microbial community, is implicated in the pathogenesis of MetS. Diets high in fat and low in fiber can induce dysbiosis, leading to an increase in pro-inflammatory bacteria and a reduction in beneficial species [12]. This microbial shift promotes systemic inflammation, a key factor in developing obesity and insulin resistance. Dysbiosis triggers immune system activation, where harmful bacteria stimulate the production of inflammatory cytokines, further contributing to insulin resistance and metabolic dysfunction [13]. The gut microbiota also plays a pivotal role in lipid metabolism and glucose regulation. Dysbiosis is associated with altered bile acid metabolism, affecting lipid absorption and storage. Additionally, the gut microbiota influences energy balance by regulating appetite-related hormones and metabolic pathways [14]. Certain gut bacteria can enhance the metabolism of dietary fats, impacting the lipid profile and either exacerbating or alleviating dyslipidemia linked to MetS. Moreover, gut bacteria affect glucose homeostasis by improving insulin sensitivity and modulating the gut-brain axis, which controls appetite and energy expenditure [15]. Intestinal permeability, often called "leaky gut," is another crucial factor in the connection between gut health and MetS. This condition arises when the gut barrier is compromised, allowing harmful substances to enter the bloodstream. Increased permeability can lead to the translocation of bacteria and their products, such as lipopolysaccharides (LPS), into the circulation, which triggers systemic inflammation-a hallmark of MetS. LPS in the bloodstream, known as endotoxemia, is associated with chronic inflammation and insulin resistance [16]. LPS, components of the outer membrane of gram-negative bacteria, are potent inducers of inflammation. Elevated LPS levels activate inflammatory pathways that impair insulin signaling, contributing to insulin resistance and metabolic dysfunction [17]. The pathophysiology of metabolic syndrome and gut dysbiosis is depicted in Figure 1.

Current supplementation therapies targeting the gut microbiome

The gut microbiome is increasingly recognized for its critical role in metabolic health, particularly in managing metabolic syndrome (MetS). Several supplementation therapies targeting the gut microbiome, such as probiotics, prebiotics, synbiotics, postbiotics, dietary fiber, and polyphenols, are being investigated for their potential benefits. These therapies operate through distinct mechanisms and have demonstrated clinical evidence supporting their efficacy in improving metabolic health [18]. Probiotics are live microorganisms that offer health benefits to the host when consumed in sufficient quantities. Common strains include Lactobacillus (e.g., *L. acidophilus*, *L. plantarum*) and Bifidobacterium (e.g., *B. bifidum*, *B. longum*). Probiotics help restore microbial balance, reduce inflammation, and improve lipid profiles. They normalize gut microbiota by outcompeting pathogenic bacteria, enhancing intestinal barrier function, and potentially lowering cholesterol levels. Clinical studies have demonstrated that specific probiotic strains can improve metabolic parameters associated with MetS, such as insulin sensitivity and waist circumference [19]. Prebiotics, however, are non-digestible food components that stimulate the growth of beneficial gut bacteria. Common prebiotics include inulin, fructooligosaccharides, and galactooligosaccharides. Prebiotics enhance the production of short-chain fatty acids (SCFAs) through fermentation by gut bacteria, which are essential for metabolic health. SCFAs help improve gut barrier function, regulate appetite, and reduce inflammation. Clinical research has shown that prebiotic supplementation can decrease risk factors for MetS, resulting in improved insulin sensitivity and reduced body fat [20]. Synbiotics combine probiotics and prebiotics to enhance their synergistic effects on gut health. This combination can improve gut microbiota diversity and metabolic outcomes more effectively than either component alone. Evidence suggests that synbiotic supplementation can enhance glucose metabolism and weight management in individuals with MetS, highlighting its potential as a therapeutic approach [21]. Postbiotics are bioactive compounds produced by probiotics, including SCFAs and microbial cell fragments. These metabolites can improve metabolic health by reducing inflammation and enhancing insulin sensitivity. Emerging research indicates that postbiotics may be promising in managing MetS, with early findings showing reductions in various metabolic risk factors [22]. Dietary fiber is crucial for promoting gut microbiota diversity and increasing SCFA production. It supports appetite regulation, lipid metabolism, and glycemic control. High-fiber diets have been shown to alleviate MetS symptoms, with clinical evidence confirming their effectiveness in improving blood glucose levels and lipid profiles [9]. Lastly, polyphenols found in foods such as fruits, vegetables, and tea interact with gut microbiota by encouraging the growth of beneficial bacteria and inhibiting pathogenic species. Polyphenols have antioxidant and anti-inflammatory properties that positively impact metabolic pathways. Studies have demonstrated that polyphenol-rich diets can lower risk factors associated with MetS, underscoring the role of nutrition in managing metabolic health [23]. Table 1 provides an overview of current supplementation therapies targeting the gut microbiome and their impact on MetS.

**Table 1:** Current supplementation therapies targeting the gut microbiome and their impact on metabolic syndrome. SCFAs: short-chain fatty acids.

Supplementation therapy	Mechanism of action	Impact on metabolic syndrome
Probiotics [24]	Introduce live beneficial bacteria to enhance gut microbial diversity and improve metabolic health.	Improves insulin sensitivity, reduces inflammation, and aids in weight management.
Prebiotics [25]	Non-digestible fibers that feed beneficial gut bacteria, promoting their growth and SCFA production.	Enhances gut barrier integrity, reduces endotoxemia, and improves lipid metabolism.
Synbiotics [26]	Combination of probiotics and prebiotics that synergistically improve gut microbiota and metabolic outcomes.	Synergistically enhances microbial diversity, reducing insulin resistance and inflammatory markers.
Postbiotics [27]	Metabolites produced by gut bacteria (e.g., SCFAs) that directly modulate immune and metabolic functions.	Reduces inflammation, improves insulin sensitivity, and strengthens gut barrier function.
Dietary fibers [25]	Increase SCFA production, improve gut barrier function, and regulate blood glucose and lipid metabolism.	Lowers cholesterol, improves glycemic control, and reduces body weight.
Polyphenols [28]	Exhibit antioxidant properties, reduce inflammation, and modulate gut microbiota composition.	Reduces oxidative stress, modulates lipid metabolism, and enhances insulin sensitivity.
Omega-3 fatty acids [29]	Anti-inflammatory properties that improve gut health reduce endotoxemia and enhance insulin sensitivity.	Decreases inflammation, improves lipid profiles, and enhances gut barrier integrity.

Fecal microbiota transplantation (FMT)

Fecal microbiota transplantation (FMT) is a therapeutic procedure designed to restore a healthy gut microbiota by transferring fecal material from a healthy donor to a recipient. This method has garnered significant interest for its potential to address gastrointestinal and metabolic disorders, including metabolic syndrome (MetS) [30]. FMT involves introducing a solution of fecal matter from a healthy donor into the recipient's intestinal tract. This procedure aims to correct dysbiosis in the recipient's gut microbiome, which can result from antibiotic use, infections, or chronic illnesses. By reintroducing a diverse and balanced microbiota, FMT seeks to restore gut health and improve related metabolic conditions [31]. The effects of FMT stem from several mechanisms, including the reduction of gut inflammation, enhancement of insulin sensitivity, and modification of microbiota composition. FMT can help modulate the immune response in the gut, leading to decreased inflammation-a key factor in many metabolic disorders [32]. Some studies suggest that FMT may improve insulin sensitivity, which could aid in managing obesity and type 2 diabetes, which are closely associated with MetS. By re-establishing a healthy microbiota, FMT can restore microbial diversity and balance, crucial for maintaining metabolic health and preventing diseases related to dysbiosis [32-36]. While FMT has demonstrated significant success in treating recurrent Clostridium difficile infections, with success rates exceeding 90% in some studies, its efficacy for other conditions, including MetS, is still being explored [37]. Preliminary trials show promise, but several challenges remain. Individual responses to FMT can vary widely and are influenced by factors such as the recipient's baseline microbiome and overall health. Additionally, there is a need for standardized protocols for donor selection, stool preparation, and administration methods to ensure consistent outcomes across studies [38]. Despite its potential, FMT's long-term efficacy and safety are crucial considerations. Although generally considered safe, potential risks include the transmission of infections and adverse reactions. Long-term monitoring of recipients is essential to detect any delayed complications. The durability of FMT's benefits on metabolic health is still uncertain, and further research is needed to assess how long the effects last and whether repeated treatments may be necessary [39].

Personalized microbiome-based approaches for MetS

Advances in gut microbiome analysis techniques have markedly improved our ability to develop personalized strategies for managing metabolic syndrome (MetS). By profiling an individual's unique microbiome composition, healthcare providers can assess their risk of developing MetS-related conditions, such as obesity, type 2 diabetes, and non-alcoholic fatty liver disease [39]. This predictive capability enables targeted interventions to prevent or delay disease onset. Personalized nutrition plays a crucial role in these microbiome-based therapies, as an individual's gut microbiome composition can influence their response to various foods and nutrients. Tailoring dietary recommendations based on microbiome profiles allows for optimizing metabolic outcomes. For example, incorporating specific prebiotics that selectively promote the growth of beneficial bacteria can enhance insulin sensitivity and reduce inflammation, leading to improved metabolic health [40]. The future of microbiome-based therapies for MetS lies in developing personalized interventions that target an individual's unique microbial composition and associated metabolic profile. This approach may involve a combination of probiotics, prebiotics, postbiotics, and fecal microbiota transplantation (FMT) to restore a healthy gut ecosystem [41]. Such precision medicine strategies promise to improve patient outcomes by providing more effective treatments tailored to individual needs. However, several challenges remain in implementing these personalized microbiome therapies in clinical practice. Individual variability in microbiome composition, the lack of standardized intervention protocols, and the need for long-term safety and efficacy data must be addressed to ensure successful outcomes. Additionally, patient adherence to personalized dietary and supplementation regimens is essential; without commitment to these tailored approaches, the potential benefits may not be fully realized [42]. Despite these challenges, the potential of personalized microbiome-based approaches for managing MetS is substantial. By leveraging an individual's unique microbial profile, healthcare providers can devise more effective and targeted interventions that prevent or delay the onset of metabolic disorders and enhance overall health and well-being [43]. As research in this field progresses, integrating microbiome profiling into routine clinical practice could revolutionize the management of metabolic syndrome and related conditions [43]. Personalized microbiome-based approaches for managing MetS are summarized in Table 2.

**Table 2:** Personalized microbiome-based approaches for managing metabolic syndrome (MetS). SCFA: short-chain fatty acid.

Approach	Description	Benefits for metabolic syndrome (MetS)
Microbiome profiling [44]	Identifying the unique composition of an individual's gut microbiota through sequencing technologies.	Helps in tailoring dietary and probiotic interventions to restore gut balance and improve metabolic health.
Targeted probiotic supplementation [45]	Administering specific strains of beneficial bacteria based on an individual's gut profile.	Enhances insulin sensitivity, reduces inflammation, and supports weight management.
Dietary modifications [46]	Personalized diets are designed to improve gut health, such as increasing fiber intake or reducing specific carbohydrates.	Improves gut microbiota composition, enhances lipid metabolism, and controls blood glucose levels.
Fecal microbiota transplantation (FMT) [47]	Transplanting gut microbiota from a healthy donor to an individual with dysbiosis.	It may improve insulin resistance, reduce inflammation, and rebalance gut microbiota in severe cases of dysbiosis.
Prebiotic-based interventions [48]	Personalized intake of prebiotics to nourish specific beneficial bacteria in the gut.	Supports gut health, increases SCFA production, and reduces endotoxemia, improving metabolic outcomes.
Microbiome-linked drug therapy [49]	Developing or administering medications that modulate the microbiome to target metabolic pathways.	Tailored therapies can target specific gut-related metabolic pathways to reduce inflammation and improve lipid and glucose metabolism.
Monitoring and feedback systems [50]	Continuous monitoring of gut microbiome changes via testing, with real-time adjustments to interventions.	Ensures personalized interventions are optimized for long-term metabolic health benefits.

Limitations and challenges of microbiome-targeted therapies

Exploring microbiome-targeted therapies for managing health conditions, particularly metabolic syndrome, faces several limitations and challenges. One major hurdle is the variability in gut microbiome composition among individuals. Each person’s microbiome is unique, shaped by a complex interplay of genetic, lifestyle, and environmental factors. This variability can result in differing responses to supplementation therapies [51]. For example, a specific probiotic strain might be highly effective for one individual but ineffective for another due to differences in their baseline microbiome. This highlights the need for personalized approaches to microbiome modulation, as a one-size-fits-all strategy may not provide optimal results for everyone [52]. Another challenge is distinguishing between the short-term and long-term efficacy of these therapies. While many studies have reported promising short-term results, questions remain regarding the sustainability of these benefits over extended periods. Maintaining a desired shift in microbiome composition often requires ongoing supplementation, which can be costly and impractical for patients. Therefore, developing strategies that induce lasting changes in the gut microbiome without continuous intervention is crucial to ensure the successful clinical application of microbiome-targeted therapies [53]. Safety and regulatory issues also pose significant challenges in this field. Unlike pharmaceuticals, many microbiome-targeted products are not subjected to rigorous safety and efficacy testing before reaching consumers. This lack of regulation raises concerns about potential adverse effects, such as dysbiosis, a condition of microbiota imbalance that can lead to further health complications. Establishing clear guidelines for developing, manufacturing, and marketing microbiome-based products is essential to ensure their safety and efficacy, protecting consumers from unregulated supplements that may not deliver the promised benefits [54]. The limitations and challenges of microbiome-targeted therapies are summarized in Table 3.

**Table 3:** Limitations and challenges of microbiome-targeted therapies. FMT: fecal microbiota transplantation.

Limitation/challenge	Description	Impact on therapy effectiveness
Inter-individual variability [55]	Significant differences in gut microbiome composition between individuals.	Limits the ability to standardize treatments and predict outcomes.
Lack of long-term data [56]	Few studies have examined the long-term effects of microbiome-targeted therapies.	Uncertain sustainability and safety of therapies over extended periods.
Complex microbiome interactions [57]	The gut microbiome interacts with diet, environment, and genetics in complex ways.	Makes it difficult to identify which specific bacteria are beneficial.
Regulatory challenges [58]	Lack of clear regulations for probiotic, prebiotic, and microbiome-based therapies.	Hinders widespread adoption and approval of therapies.
Inconsistent clinical outcomes [59]	Microbiome-targeted therapies may show varying effectiveness in different individuals.	Reduces the generalizability of research findings to broader populations.
Limited understanding of microbiome functions [60]	Many microbiome species and their functions are not yet fully understood.	Impedes precise targeting of therapies and understanding of mechanisms.
High cost and accessibility [61]	Advanced microbiome sequencing and personalized therapies can be expensive.	Limits access to therapies, especially in low-resource settings.
Risk of adverse effects [62]	Altering the microbiome could potentially lead to unintended consequences, such as dysbiosis.	Raises safety concerns and limits the widespread application of therapies.
Ethical concerns [63]	Ethical issues surrounding the manipulation of gut microbiota, especially with techniques like FMT.	Requires careful consideration of risks and benefits.

Future directions and emerging research

Exploring microbiome-targeted therapies for managing health conditions, particularly metabolic syndrome, presents several limitations and challenges. One significant obstacle is the variability in gut microbiome composition among individuals [64-66]. Each person’s microbiome is unique and influenced by genetic, lifestyle, and environmental factors [67-70]. This variability can lead to differing responses to supplementation therapies [51]. For instance, a specific probiotic strain may be highly effective for one person but ineffective for another due to differences in their baseline microbiome. This underscores the need for personalized approaches to microbiome modulation, as a one-size-fits-all strategy may not yield optimal results for everyone [52]. Another challenge lies in distinguishing between these therapies' short-term and long-term efficacy. Although many studies have shown promising short-term outcomes, questions persist about the sustainability of these benefits over time. Achieving and maintaining a desired shift in microbiome composition often requires ongoing supplementation, which can be costly and impractical for patients. Consequently, developing strategies that induce lasting changes in the gut microbiome without continuous intervention is crucial for the successful clinical application of microbiome-targeted therapies [53]. Safety and regulatory issues also pose significant challenges. Unlike pharmaceuticals, many microbiome-targeted products are not subjected to rigorous safety and efficacy testing before reaching consumers. This lack of regulation raises concerns about potential adverse effects, such as dysbiosis, a condition characterized by an imbalance in the microbiota that can lead to further health complications. Establishing clear guidelines for developing, manufacturing, and marketing microbiome-based products is essential to ensure their safety and efficacy, protecting consumers from unregulated supplements that may not deliver the promised benefits [54]. These limitations and challenges associated with microbiome-targeted therapies are summarized in Table 4.

**Table 4:** Future directions and emerging research in microbiome-targeted therapies for metabolic syndrome (MetS).

Future direction/emerging research	Description	Potential impact on metabolic syndrome (MetS)
Advanced microbiome profiling [70]	Utilizing next-generation sequencing and metagenomics to better understand individual microbiomes.	Enables highly personalized therapies with greater precision for treating MetS.
Synthetic microbiomes [71]	Developing synthetic or engineered microbial communities to restore gut balance.	Potential to create targeted microbial communities that can be customized for metabolic conditions.
Microbiome-based drug development [72]	Creating drugs that modulate the microbiome or are derived from microbial metabolites.	Offers novel therapeutic options for managing insulin resistance, lipid metabolism, and inflammation in MetS.
Precision prebiotics and probiotics [48]	Designing specific strains of probiotics and tailored prebiotics that target key metabolic pathways.	Improved effectiveness in managing obesity, insulin sensitivity, and gut dysbiosis in individuals with MetS.
Gut-brain axis research [73]	Exploring how the gut microbiome influences neuroendocrine pathways related to appetite, mood, and energy balance.	This could lead to new strategies to address metabolic diseases by targeting gut-brain interactions.
Microbiota transplant innovations [74]	Developing safer and more effective techniques for fecal microbiota transplantation (FMT) and synthetic alternatives.	Provides options for severe dysbiosis and metabolic disorders, with better control over microbiota composition.
Artificial intelligence in microbiome research [75]	Using AI and machine learning to analyze large microbiome datasets and predict therapeutic outcomes.	Enhances the ability to tailor therapies and predict patient responses, leading to more successful interventions.
Microbiome as a biomarker [76]	Identifying specific microbiome signatures as biomarkers for early detection of MetS and related conditions.	Improves early diagnosis and personalized prevention strategies.
Longitudinal studies on microbiome interventions [77]	Conducting long-term studies to evaluate the sustainability and safety of microbiome-targeted therapies.	Provides essential data for ensuring the efficacy and safety of these therapies over time.

## Conclusions

In recent years, the relationship between the gut microbiome and metabolic syndrome (MetS) has garnered increasing attention as a potential avenue for therapeutic intervention. The gut microbiota plays a critical role in regulating key metabolic processes, and its imbalance, or dysbiosis, has been strongly linked to the development of insulin resistance, inflammation, and other hallmarks of MetS. Through the modulation of the gut microbiome via supplementation therapies such as probiotics, prebiotics, synbiotics, and postbiotics, there is growing evidence that these interventions can positively influence metabolic health by improving glucose metabolism and lipid profiles and reducing systemic inflammation. However, while promising, this field of research remains in its early stages. More comprehensive clinical trials are needed to fully understand microbiome-targeted therapies' long-term efficacy and safety in managing MetS. As we continue to explore the therapeutic potential of these interventions, the gut microbiome presents a novel and promising target for mitigating the global burden of MetS and its associated complications.

## References

[1] Rochlani Y, Pothineni NV, Kovelamudi S, Mehta JL (2017). Metabolic syndrome: pathophysiology, management, and modulation by natural compounds. Ther Adv Cardiovasc Dis.

[2] Swarup S, Ahmed I, Grigorova Y, Zeltser R (2024). Metabolic syndrome. StatPearls (Internet).

[3] Cornier MA, Dabelea D, Hernandez TL (2008). The metabolic syndrome. Endocr Rev.

[4] Saklayen MG (2018). The global epidemic of the metabolic syndrome. Curr Hypertens Rep.

[5] Chan SM, Selemidis S, Bozinovski S, Vlahos R (2019). Pathobiological mechanisms underlying metabolic syndrome (MetS) in chronic obstructive pulmonary disease (COPD): clinical significance and therapeutic strategies. Pharmacol Ther.

[6] Clemente-Suárez VJ, Martín-Rodríguez A, Redondo-Flórez L, López-Mora C, Yáñez-Sepúlveda R, Tornero-Aguilera JF (2023). New insights and potential therapeutic interventions in metabolic diseases. Int J Mol Sci.

[7] Thursby E, Juge N (2017). Introduction to the human gut microbiota. Biochem J.

[8] Rinninella E, Raoul P, Cintoni M, Franceschi F, Miggiano GAD, Gasbarrini A, Mele MC (2019). What is the healthy gut microbiota composition? A changing ecosystem across age, environment, diet, and diseases. Microorganisms.

[9] den Besten G, van Eunen K, Groen AK, Venema K, Reijngoud DJ, Bakker BM (2013). The role of short-chain fatty acids in the interplay between diet, gut microbiota, and host energy metabolism. J Lipid Res.

[10] Wu HJ, Wu E (2012). The role of gut microbiota in immune homeostasis and autoimmunity. Gut Microbes.

[11] Dabke K, Hendrick G, Devkota S (2019). The gut microbiome and metabolic syndrome. J Clin Invest.

[12] Wang PX, Deng XR, Zhang CH, Yuan HJ (2020). Gut microbiota and metabolic syndrome. Chin Med J (Engl).

[13] Scheithauer TPM, Rampanelli E, Nieuwdorp M, Vallance BA, Verchere CB, van Raalte DH, Herrema H (2020). Gut microbiota as a trigger for metabolic inflammation in obesity and type 2 diabetes. Front Immunol.

[14] Yu Y, Raka F, Adeli K (2019). The role of the gut microbiota in lipid and lipoprotein metabolism. J Clin Med.

[15] Schoeler M, Caesar R (2019). Dietary lipids, gut microbiota and lipid metabolism. Rev Endocr Metab Disord.

[16] Zhang Y, Zhu X, Yu X, Novák P, Gui Q, Yin K (2023). Enhancing intestinal barrier efficiency: a novel metabolic diseases therapy. Front Nutr.

[17] Page MJ, Kell DB, Pretorius E (2022). The role of lipopolysaccharide-induced cell signalling in chronic inflammation. Chronic Stress.

[18] Antony MA, Chowdhury A, Edem D (2023). Gut microbiome supplementation as therapy for metabolic syndrome. World J Diabetes.

[19] Fijan S (2014). Microorganisms with claimed probiotic properties: an overview of recent literature. Int J Environ Res Public Health.

[20] Davani-Davari D, Negahdaripour M, Karimzadeh I (2019). Prebiotics: definition, types, sources, mechanisms, and clinical applications. Foods.

[21] Markowiak P, Śliżewska K (2017). Effects of probiotics, prebiotics, and synbiotics on human health. Nutrients.

[22] Hijová E (2024). Postbiotics as metabolites and their biotherapeutic potential. Int J Mol Sci.

[23] Plamada D, Vodnar DC (2021). Polyphenols—gut microbiota interrelationship: a transition to a new generation of prebiotics. Nutrients.

[24] Wang X, Zhang P, Zhang X (2021). Probiotics regulate gut microbiota: an effective method to improve immunity. Molecules.

[25] Nogal A, Valdes AM, Menni C (2021). The role of short-chain fatty acids in the interplay between gut microbiota and diet in cardio-metabolic health. Gut Microbes.

[26] Jiang H, Cai M, Shen B, Wang Q, Zhang T, Zhou X (2022). Synbiotics and gut microbiota: new perspectives in the treatment of type 2 diabetes mellitus. Foods.

[27] Rafique N, Jan SY, Dar AH (2023). Promising bioactivities of postbiotics: a comprehensive review. Agric Food Res.

[28] Shabbir U, Tyagi A, Elahi F, Aloo SO, Oh DH (2021). The potential role of polyphenols in oxidative stress and inflammation induced by gut microbiota in Alzheimer’s disease. Antioxidants (Basel).

[29] Costantini L, Molinari R, Farinon B, Merendino N (2017). Impact of omega-3 fatty acids on the gut microbiota. Int J Mol Sci.

[30] Gupta S, Allen-Vercoe E, Petrof EO (2016). Fecal microbiota transplantation: in perspective. Therap Adv Gastroenterol.

[31] Biazzo M, Deidda G (2022). Fecal microbiota transplantation as new therapeutic avenue for human diseases. J Clin Med.

[32] Chen L, Guo L, Feng S (2023). Fecal microbiota transplantation ameliorates type 2 diabetes via metabolic remodeling of the gut microbiota in db/db mice. BMJ Open Diabetes Res Care.

[33] Wu Z, Zhang B, Chen F (2023). Fecal microbiota transplantation reverses insulin resistance in type 2 diabetes: a randomized, controlled, prospective study. Front Cell Infect Microbiol.

[34] Zhang J, Wang H, Liu Y, Shi M, Zhang M, Zhang H, Chen J (2024). Advances in fecal microbiota transplantation for the treatment of diabetes mellitus. Front Cell Infect Microbiol.

[35] Wang H, Lu Y, Yan Y (2020). Promising treatment for type 2 diabetes: fecal microbiota transplantation reverses insulin resistance and impaired islets. Front Cell Infect Microbiol.

[36] Zhou X, Chen R, Cai Y, Chen Q (2024). Fecal microbiota transplantation: a prospective treatment for type 2 diabetes mellitus. Diabetes Metab Syndr Obes.

[37] Davidovics ZH, Michail S, Nicholson MR (2019). Fecal microbiota transplantation for recurrent Clostridium difficile infection and other conditions in children: a joint position paper from the North American Society for Pediatric Gastroenterology, Hepatology, and Nutrition and the European Society for Pediatric Gastroenterology, Hepatology, and Nutrition. J Pediatr Gastroenterol Nutr.

[38] Bibbò S, Settanni CR, Porcari S, Bocchino E, Ianiro G, Cammarota G, Gasbarrini A (2020). Fecal microbiota transplantation: screening and selection to choose the optimal donor. J Clin Med.

[39] Wardill HR, Secombe KR, Bryant RV, Hazenberg MD, Costello SP (2019). Adjunctive fecal microbiota transplantation in supportive oncology: emerging indications and considerations in immunocompromised patients. EBioMedicine.

[40] Durack J, Lynch SV (2019). The gut microbiome: relationships with disease and opportunities for therapy. J Exp Med.

[41] Gulliver EL, Young RB, Chonwerawong M (2022). Review article: the future of microbiome-based therapeutics. Aliment Pharmacol Ther.

[42] Wang RC, Wang Z (2023). Precision medicine: disease subtyping and tailored treatment. Cancers (Basel).

[43] Shapiro H, Suez J, Elinav E (2017). Personalized microbiome-based approaches to metabolic syndrome management and prevention. J Diabetes.

[44] Hills RD Jr, Pontefract BA, Mishcon HR, Black CA, Sutton SC, Theberge CR (2019). Gut microbiome: profound implications for diet and disease. Nutrients.

[45] Salles BIM, Cioffi D, Ferreira SRG (2020). Probiotics supplementation and insulin resistance: a systematic review. Diabetol Metab Syndr.

[46] Cronin P, Joyce SA, O'Toole PW, O'Connor EM (2021). Dietary fibre modulates the gut microbiota. Nutrients.

[47] Fang H, Yao T, Li W (2023). Efficacy and safety of fecal microbiota transplantation for chronic insomnia in adults: a real world study. Front Microbiol.

[48] Ji J, Jin W, Liu SJ, Jiao Z, Li X (2023). Probiotics, prebiotics, and postbiotics in health and disease. MedComm.

[49] Olofsson LE, Bäckhed F (2022). The metabolic role and therapeutic potential of the microbiome. Endocr Rev.

[50] Ngashangva L, Chattopadhyay S (2023). Biosensors for point-of-care testing and personalized monitoring of gastrointestinal microbiota. Front Microbiol.

[51] Yadav M, Chauhan NS (2022). Microbiome therapeutics: exploring the present scenario and challenges. Gastroenterol Rep (Oxf).

[52] Hou Q, Zhao F, Liu W (2020). Probiotic-directed modulation of gut microbiota is basal microbiome dependent. Gut Microbes.

[53] Leeming ER, Johnson AJ, Spector TD, Le Roy CI (2019). Effect of diet on the gut microbiota: rethinking intervention duration. Nutrients.

[54] Dwyer JT, Coates PM, Smith MJ (2018). Dietary supplements: regulatory challenges and research resources. Nutrients.

[55] Schupack DA, Mars RA, Voelker DH, Abeykoon JP, Kashyap PC (2022). The promise of the gut microbiome as part of individualized treatment strategies. Nat Rev Gastroenterol Hepatol.

[56] Gupta A, Saha S, Khanna S (2020). Therapies to modulate gut microbiota: past, present and future. World J Gastroenterol.

[57] Boccuto L, Tack J, Ianiro G, Abenavoli L, Scarpellini E (2023). Human genes involved in the interaction between host and gut microbiome: regulation and pathogenic mechanisms. Genes (Basel).

[58] Thanush D, Basavaraj HC, Gowrav MP (2023). Current regulation and initial considerations for successful development and commercialization of microbiome therapies. AGMR.

[59] DeVeaux A, Ryou J, Dantas G, Warner BB, Tarr PI (2023). Microbiome-targeting therapies in the neonatal intensive care unit: safety and efficacy. Gut Microbes.

[60] Aggarwal N, Kitano S, Puah GRY, Kittelmann S, Hwang IY, Chang MW (2023). Microbiome and human health: current understanding, engineering, and enabling technologies. Chem Rev.

[61] Goetz LH, Schork NJ (2018). Personalized medicine: motivation, challenges, and progress. Fertil Steril.

[62] Hrncir T (2022). Gut microbiota dysbiosis: triggers, consequences, diagnostic and therapeutic options. Microorganisms.

[63] Ma Y, Chen H, Lan C, Ren J (2018). Help, hope and hype: ethical considerations of human microbiome research and applications. Protein Cell.

[64] Abouelela ME, Helmy YA (2024). Next-generation probiotics as novel therapeutics for improving human health: current trends and future perspectives. Microorganisms.

[65] Mousavinasab F, Karimi R, Taheri S (2023). Microbiome modulation in inflammatory diseases: progress to microbiome genetic engineering. Cancer Cell Int.

[66] Santos-Beneit F (2024). What is the role of microbial biotechnology and genetic engineering in medicine?. Microbiologyopen.

[67] Araujo R, Borges-Canha M, Pimentel-Nunes P (2022). Microbiota modulation in patients with metabolic syndrome. Nutrients.

[68] Strasser B, Wolters M, Weyh C, Krüger K, Ticinesi A (2021). The effects of lifestyle and diet on gut microbiota composition, inflammation and muscle performance in our aging society. Nutrients.

[69] Manrique P, Montero I, Fernandez-Gosende M, Martinez N, Cantabrana CH, Rios-Covian D (2024). Past, present, and future of microbiome-based therapies. Microbiome Res Rep.

[70] Zhang X, Li L, Butcher J, Stintzi A, Figeys D (2019). Advancing functional and translational microbiome research using meta-omics approaches. Microbiome.

[71] van Leeuwen PT, Brul S, Zhang J, Wortel MT (2023). Synthetic microbial communities (SynComs) of the human gut: design, assembly, and applications. FEMS Microbiol Rev.

[72] Crudele L, Gadaleta RM, Cariello M, Moschetta A (2023). Gut microbiota in the pathogenesis and therapeutic approaches of diabetes. EBioMedicine.

[73] Carabotti M, Scirocco A, Maselli MA, Severi C (2015). The gut-brain axis: interactions between enteric microbiota, central and enteric nervous systems. Ann Gastroenterol.

[74] Sahle Z, Engidaye G, Shenkute Gebreyes D, Adenew B, Abebe TA (2024). Fecal microbiota transplantation and next-generation therapies: a review on targeting dysbiosis in metabolic disorders and beyond. SAGE Open Med.

[75] Novielli P, Romano D, Magarelli M (2024). Explainable artificial intelligence for microbiome data analysis in colorectal cancer biomarker identification. Front Microbiol.

[76] Hajjo R, Sabbah DA, Al Bawab AQ (2022). Unlocking the potential of the human microbiome for identifying disease diagnostic biomarkers. Diagnostics (Basel).

[77] Hitch TCA, Hall LJ, Walsh SK (2022). Microbiome-based interventions to modulate gut ecology and the immune system. Mucosal Immunol.

